# Risk of HIV and associated factors among infants born to HIV positive women in Amhara region, Ethiopia: a facility based retrospective study

**DOI:** 10.1186/1756-0500-7-876

**Published:** 2014-12-04

**Authors:** Zelalem Berhan, Fantu Abebe, Molla Gedefaw, Mulugeta Tesfa, Muluken Assefa, Yilkal Tafere

**Affiliations:** Senior Clinical Mentor, Management Science for Health (MSH), Bahir Dar, Ethiopia; Education and Training Officer for PSE of health cadres, P.O. Box:1566, Bahir Dar, Ethiopia; GAMBY College of Health Science, Bahir Dar, Ethiopia; College of Medicine and Health Science, Debremarkos University, Debremarkos, Ethiopia; Bahir Dar Health Science College, Bahir Dar, Ethiopia; Debretabor Health Science College, Debretabor, Ethiopia

**Keywords:** HIV exposed infants, DBS, DNA/PCR

## Abstract

**Background:**

The estimated HIV prevalence among pregnant women in Ethiopia is 1.2 percent and unfortunately one of every 3 children born to these women gets infected with HIV. Elimination of these mother-to-child transmissions (MTCT) of HIV is possible through HIV testing during pregnancy and taking antiretroviral medications. However, only 24 percent of the pregnant women living with HIV have yet received the medication needed to prevent the transmission of HIV. Hence, there exists a concern that the rate of HIV infection among infants born to HIV positive mothers is increasing. This study assessed the prevalence of HIV infection and associated factors among infants born to women living with HIV, in South Gondar zone, Amhara region, Ethiopia.

**Methods:**

Facility based document review was conducted upon 434 charts. The study participants were HIV exposed infants enrolled from January to December 2012. The data were reviewed from all the 17 health facilities which were providing PMTCT services in the zone. The study included 434 HIV exposed infants having an HIV Deoxyribonucleic Acid (DNA) Polymerase Chain Reaction (PCR) test result. The data were collected using structured data extraction tool. Binary logistic regression analysis was employed to assess the putative association of independent variables with the outcome variable. Significance was taken at a P value <0.05 and 95% confidence level.

**Result:**

The prevalence of HIV among HIV exposed infants was 10.1% (95% CI = 7.3 - 13%). Delayed diagnosis (AOR = 2.7, 95% CI = 1.3, 29.4), mixed infant feeding (AOR = 8.8, 95% CI = 4.5, 22.8), failure to receive either antiretroviral therapy or prophylaxis during pregnancy or breast feeding (AOR = 21.6, 95% CI = 14.5, 39.8) and shorter duration of HIV treatment (AOR = 12, 95% CI = (4.2, 45.0) were the factors that increase the risk of mother- to- child transmission of HIV.

**Conclusion:**

The prevalence of HIV infection among HIV exposed infants is strikingly high. Inadequate use of antiretroviral therapy and skilled delivery care were the factors that enhance mother-to-child transmission of HIV. Integrated and audience specific education and promotion for seeking obstetric care and HIV services is instrumental to curb the devastating consequences of HIV on pregnant women and their newborns

## Background

Globally, about 330,000 children were infected with HIV in 2011, with over 90% of these infections occurring in sub-Sahara Africa, and mainly through mother-to-child transmission [1]. Twenty two countries account for more than 90% of the global burden. Ethiopia is one of these priority countries where one in every 3 children born to a woman living with HIV gets infected with HIV [1–3].

Disease progression is aggressive if infants acquire the infection before or during the time of delivery [4]. If left untreated, almost half of these children will die before turning to second year, and 75% of them die by the age of five years. Most of these deaths in children with HIV could have been avoided through early infant diagnosis (EID) and provision of effective care and treatment. Interventions like the use of Antiretroviral (ART) drugs by infected pregnant women, safe delivery practices and safe infant feeding have helped reduce the risk of transmission to infants (from 40%to 5%) [1, 5].

In Ethiopia an estimated 1.2 percent of pregnant women are living with HIV. Consequently, one of every 3 children born to these women is being infected with HIV [1, 2]. Elimination of these mother-to-child transmissions (MTCT) of HIV would be possible through HIV testing during pregnancy and taking antiretroviral medications. Hence, the government of Ethiopia has been expanding various preventive measures and services against this Mother-To-Child Transmission (PMTCT). To combat infections among infants and keep mothers alive, a comprehensive package of interventions, including provision of appropriate HIV treatment, and care and support services have been scaled up across the regions in Ethiopia [6].

Moreover, since March 2013 all HIV positive pregnant women in Ethiopia, perhaps in the region, have been eligible to start long term antiretroviral therapy (ART) through a transition to a new treatment package known as option B+. Implementing this package is a critical step towards eliminating at least 98% of these mother-to-child transmissions of HIV [5, 6].

Despite the availability and scale up of these life-saving interventions, only 24% of pregnant women living with HIV in the region have been receiving the medication to prevent mother-to-child transmission of HIV [1, 3]. Moreover, among women who utilize skilled delivery services (12%) [2], most often go untested for HIV, with devastating consequences for themselves and their newborns. As a result of this trend, there is a growing concern that the risk of mother-to-child transmission of HIV in the region, perhaps in the south Gondar zone, Amhara may be increasing [1, 3].

Early diagnosis of infections among infants with HIV followed by prompt ART treatment can help reduce morbidity and mortality among them. Failing to do this will usually lead to rapid disease progression and death. Almost 50% of the infants infected during pregnancy or delivery died within their one year age, while about 50% of the children infected during breastfeeding died within nine years of the infection [6, 7].

In infants, antibody testing to diagnose HIV infection is inadequate, as the passively acquired maternal antibodies in the infant may yield false-positive results for up to 18 months or longer. It is, therefore, important to provide accurate diagnostic services for identifying of infants infected with HIV. Because of its high sensitivity and specificity, DNA polymerase chain reaction (PCR) has been widely used for diagnosis of HIV amongst exposed infants. This technology involves the use of a small spot taken as a Dried Blood Spot (DBS) sample, and can also be employed for identification of infection at birth [7]. This is a molecular test service, which has been successfully implemented in major regions in Ethiopia. We only enrolled those HIV exposed and DBS tested infants in the zone.

In Ethiopia, few studies [7, 8] tried to demonstrate the risk of mother-to-child transmission of HIV. However, they were either confined to a single facility or assessed the risk among urban dwellers only or used relatively remote data. Consequently, assessing the risk and determinant factors in the entire zonal administration that involve both urban and rural families through enrolling all health facilities that provide PMTCT service and using relatively latest data available would, however, justify the importance of this research. In this study, we aimed to determine the prevalence and associated factors of HIV infection among HIV exposed infants in South Gondar zone, Amhara region, Ethiopia.

## Methods

A facility based cross-sectional study design was employed from January to June 2013 to collect the data from the registration and follow up log books in the PMTCT and HIV exposed infants’ (HEI) follow-up units.

This study was conducted in South Gondar zone, which is one of the 11 administrative zones in Amhara National Regional State, Northwest Ethiopia. Amhara region is the second largest and most populous regional state in Ethiopia. It has a total of 19.2 million people. It is one of the hardest HIV/AIDS-hit regions in the country. The prevalence of HIV among adults and pregnant women in the region is 1.5% and 0.8% respectively [2]. The south Gondar administrative zone is inhabited by about 2.4 million people, and is administratively divided into 12 districts and 5 town administrations [9]. The burden of HIV in the zone, if not worst, may not be different from the regional estimate.

This zone has one general hospital and 16 health centers, all of which are providing PMTCT services. The study was conducted in all of these facilities. The data collection was conducted from January to June 2013.

The study population involved HIV-exposed infants enrolled in all health facilities providing PMTCT services. The data were extracted from all registered HIV exposed and DNA/ PCR tested infant charts documented from January 1^st^ to 31^st^ December 2012.

Records were considered eligible for inclusion if an infant’s age at diagnosis was recorded. They were also considered eligible for inclusion if both infant and maternal HIV treatment and prophylaxis data are available, and if information is available on infant feeding practice, place of delivery and DNA/PCR test result. Based on this notion, 434 charts were identified and enrolled in the study.

DNA/PCR test result was the dependent variable, while characteristics that might determine the risk of HIV infection such as time of HIV testing, intake of prophylaxis by infants, maternal ART or ARV prophylaxis intake, place of delivery, infant feeding practice, maternal education, monthly income and duration of treatment were the independent variables.

A structured data extraction tool was developed, pretested and used to collect the information from the charts. The tool was adapted from the national standard HIV exposed infant follow up formats and PMTCT registration log book, which comprises, socio-demographic characteristics, information on ARV prophylaxis for the mother and infant, place of delivery and infant feeding practice and some other important variables. One ART trained nurse and one ART trained health officer or midwife in each health facility were recruited to collect the data.

Training was given for data collectors and supervisors one day. The collected data were reviewed daily for completeness, accuracy, clarity, and consistency by the supervisors and the principal investigators. Then codes were given to the completed questionnaires. The data were cleaned and edited before being entered into EPI-info version 3.5.2. After the data were checked for correct entry into EPI-info, they were exported into SPSS version 16 software for analysis. Further data cleaning and frequency run was made to check for accuracy, outliers, consistencies and missed values and variables. Any error identified was corrected.

Frequency run, cross tabulations and summary statistics were used to describe the study population in relation to relevant variables. Crude and adjusted odds ratios were computed to assess presence of association and the magnitude of statistical significance. A *P*-value of less than 0.05 at 95% CI data was considered statistically significant. Binary logistic regression analysis was employed to determine the effect of each independent variable on the outcome variable and to control for the effect of confounding.

Ethical clearance was obtained from Debre Markos University ethical clearance committee. Official letter was also sought from Amhara Regional State Health Bureau and South Gondar zonal Health department. The zonal health department and each district health office offered letters to inform individuals in charge of all eligible health facilities. The data retrieved from each health center’s HIV exposed infant records and PMTCT registration log books were entirely used anonymously and the files used did not bear any name or identification number. The collected data were kept strictly confidential.

## Result

Among the total 455 reviewed HIV exposed infants’ (HEI) and HIV positive pregnant women's records, 434 charts were found to have complete information and then enrolled for the study.

### Socio-demographic characteristics of the study participants

Female HEI infants constitute 51.6%, whereas the remaining 48.4% were males. Age at the time of diagnosis for the majority (58.8%) of infants was between 6 weeks and 6 months of age.

Majority (85.9%) of the women were married at the time of enrollment in HIV care and support service. More than half (66.8%) of the enrolled HIV positive pregnant women were unable to read and write. In addition, majority (57.8%) of the mothers were housewives in occupation.

### Findings on HIV care and support

Nearly 93% of HIV positive women were enrolled in HIV care and support service during the last pregnancy period. Among these enrolled 402 HIV positive pregnant women, 63.4% were taking highly active ART and 30.3% were put on ARV prophylaxis. Three hundred twenty eight (75.6%) infants were born in health facilities where skilled delivery care was available. Moreover, 351 (81%) of these newborns were given ARV prophylaxis right after birth. Pertaining to the duration of ART treatment a pregnant woman received, 338 (89.7%) mothers had received the treatment for longer than four weeks (Table 1).

**Table 1:** **Socio-demographic characteristics of HIV exposed infants and their mothers, South Gondar, Amhara, Ethiopia (n = 434), July 2013**

Variables	Number	Percentage
**Sex of the Infant**		
Male	210	48.4
Female	224	51.6
**Age of the mother**		
15-24	95	21.9
25-34	260	59.9
> = 35	79	18.4
**Marital status of the mother**		
Currently married	373	85.9
Currently not married	61	14.1
**Educational status of the mother**		
Unable to read and write	251	57.8
Able to read and write	183	42.2
**Occupational status of mother**		
House wife	290	66.8
Governmental employed	22	5.1
Unemployed	122	28.1
**Number of children a mother has**		
1-3	369	85
4 and above	65	15

The majority (351 or 80.9%) of the newborns born to HIV positive women were given ARV prophylaxis accordingly, however, 83 (19.1%) did not receive any prophylaxis at all. Among infants who received ARV prophylaxis, the majority (141 or 40.2%) had taken single dose Nevirapin plus AZT for seven days. The remaining 106 (30.2%), 48 (13.7%), 42 (12%) and 14 (4%) infants received daily Nevirapin for 45 days, single dose Nevirapin, daily Nevirapin until cessation of breastfeeding and single dose Nevirapin plus AZT for 28 days, respectively.

Pertaining to breast feeding, 402 (92.6%) infants were given exclusive breastfeeding, and the remaining 32 (7.4%) was put on mixed feeding. At the time of the study, 390(89.9%) infants were on active follow up, whereas 32 (7.4%) children were lost to follow up services and 12 (2.8%) children reportedly died.

With regard to the time of diagnosis of infants born to HIV positive mothers, the majority (255 or 58.8%) had their DBS tested between 6 weeks and 6 months. While only 110 (25.3%) infants were tested at the right time, which was at 6 weeks and the remaining 69 (15.9%) had their DBS test after 6 months (Table 2). The mean age at which DBS test done was 17 weeks.

**Table 2:** **Findings on HIV care and support of HIV exposed infants and their mothers, South Gondar, Amhara, Ethiopia (n = 434), July 2013**

Variables	Number	Percentage
**Infant’s age at DBS testing**		
At 6 weeks	110	25.3
6 weeks to 6 months	255	58.8
After 6 months	69	15.9
**Infants Received CPT at 6 weeks of birth**		
Yes	377	86.9
No	57	13.1
**Infants Received ARV prophylaxis at birth**		
Yes	351	80.9
No	83	19.1
**Infants feeding practice**		
Exclusive Breast feeding	402	92.6
Replacement feeding	0	0
Mixed feeding	32	7.4
**Mothers enrolled in HIV/ART care**		
Yes	402	92.6
No	32	7.4
**Mother received ART/ARV prophylaxis (n = 402)**		
ART	255	63.4
ARV	122	30.3
None	25	6.2

### Result of HIV testing among HEIs

**Figure 1:**
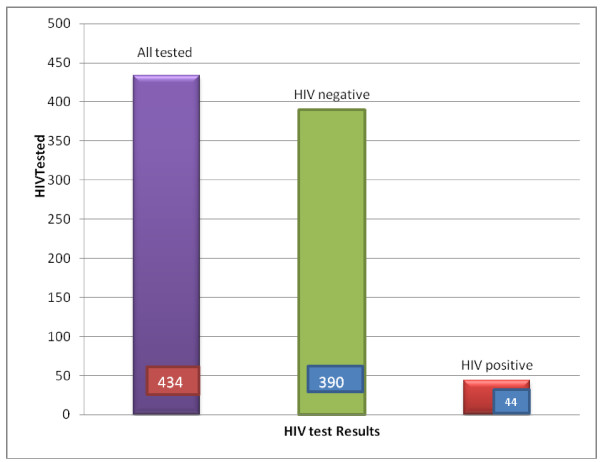
**DNA/ PCR Test results of HEI, South Gondar Zone, Amhara, 2013.**

The prevalence of HIV infection among the infants born to HIV positive mothers was 10.1% (44 out of 434 infants) (Figure 1), with a 95% confidence interval between 7.3% and 13%.

### Bivariate analysis of factors associated with DNA/PCR result

Infants who got DBS tests after 6 months of their age were more likely to have positive test results than their counterparts (COR = 9.24, 95% CI = (3.5, 24.2)). Infants born to mothers in the age range between 25–34 years were more likely to have positive DNA/PCR test results; however, it has no statistically significant association (COR = 1. 4, 95% CI = 0. 6, 4.9).

Infants their DBS tested after 6 months were more likely to have positive HIV results than their counterparts (COR = 9.24, 95% CI = (3.5, 24.2)). Infants born to mothers in the age range between 25–34 years were more likely to have higher odds of positive DNA/PCR positivity than other age categories; however, it has no statistically significant association (COR = 1. 4, 95% CI = 0. 6, 4.9) with the outcome variable.

On the other hand, infants born to mothers with no education were also more likely to be DNA/PCR positive than infants born from educated mothers (COR = 8.4, 95% CI = 2.9, 14.8). Also, all DNA/PCR positive infants were born to mothers having few (1–3) numbers of children. Besides, infants who did not receive ARV prophylaxis or ART at all were more likely to be HIV positive (COR = 10.6, 95% CI = 3.5, 13.8), but no difference was observed between taking either ARV or ART and HIV test result.

Those infants who received mixed feeding in the first six months of their life time were more likely to be DNA/PCR positive than those children who only received exclusive breastfeeding (COR = 12. 4, 95% CI = 6.0, 18.1). Meanwhile, mothers who were not enrolled in HIV care and support service had relatively higher risks of giving birth to HIV HIV positive infants than their counterparts (COR = 6. 9, 95% CI = (3.1, 15.4).

Infants born to mothers who received neither ART nor ARV prophylaxis were more likely than their counterparts to have positive result (COR = 16.0, 95% CI = (6.8, 24.9). Moreover, those infants whose mothers had taken ART or ARV prophylaxis for a duration of less than 4 weeks were more likely to be DNA/PCR positive (COR = 23.8, 95% CI = 8.8, 39.0). Lastly, infants born at home were more likely to be DNA/PCR positive than those born at health facilities (COR = 8.8, 95% CI = (4.4, 17.5) (Table 3).

**Table 3:** **Bivariate Analysis of factors associated with HIV positivity, South Gondar, Amhara, Ethiopia July 2013**

	DBS Result		COR (95% CI)	***P***value
	Negative	Positive		
**Age at DBS tested**				
At 6 week	104	6	1	0.002
6^th^ week to 6 months	241	14	1.00 (0.37, 2.69)	
After 6 months	45	24	9.24 (3.5,24.2)*	
**Mothers’ Age**				
15-24	83	12	1	0.761
25-34	234	26	1.7 (0.6, 4.6)	
35+	73	6	1.3 (0.5, 3.4)	
**Maternal Education**				
Unable read & write	211	40	8.4 (2.9, 14.8)*	0.013
Able to read & write	179	4	1	
**Infant ARV prophylaxis**				
Yes	337	14	1	0.0001
No	53	30	10.6 (3.5, 13.8)*	
**Place of delivery**				
Health facility	314	14	**1**	0.0001
Home	76	30	8.8 (4.4, 17.5)*	
**Feeding practice**				
EBF	374	28	1	0.0001
MF	16	16	12.4 (6.0, 18.1)*	
**Infants enrolled for HIV care & support**				
Yes	370	32	1	0.004
No	20	12	6.7 (3.1, 15.4)*	
**Mothers’ treatment**				
ART	245	10	1	0.0001
ARV prophylaxis	110	12	2.6 (1.1, 6.3)*	
None	15	10	16.0 (6.8, 24.9)*	
**Duration of treatment**				
>4 weeks	330	8	**1**	0.0001
<=4 weeks	25	14	23 (8.8, 39)*	

*Statistically significant at *P* value less than 0.05 and 95% CI.

### Results of multivariate analysis

We entered the variables that had a *P* value < 0.2 in the bivariate analysis (crude) to the final multivariate (adjusted) analysis model.

To begin with, those infants who took DBS tests after 6 months of their age were more likely to have positive DNA/PCR results (AOR = 1. 3,95% CI = 1.1, 10.1) than those tested in the earlier ages. On the other hand, infants who did not receive any prophylaxis were 6.7 times higher to become HIV positive than protected children (AOR = 6.7, 95% CI = 2.9, 23.1). Mixed feeding was also significantly associated with HIV positive test results (AOR = 8.8, 95% CI = 4.5, 22.8). Moreover, infants born to women who did not receive either ART or ARV prophylaxis during pregnancy or breastfeeding were found to have a higher likelihood of contracting HIV from their mothers. Similarly, infants born to mothers who did not receive ART or ARV prophylaxis at all or who received it for less than 4 weeks, were found to be with increased likelihood (21.6% and 12%, respectively ) of being HIV positive (AOR = 21.6,95% CI = 14.5, 39.8) (AOR = 12, 95% CI = (4.2, 45) (Table 4).

**Table 4:** **Multivariate Analysis of factors associated with HIV positivity, South Gondar Amhara, Ethiopia July 2013**

Variables	DBS Result		COR (95% CI)	AOR (95% CI)
	Negative	Positive		
**Age at DBS tested**				
At 6 week	104	6	1	1
6^th^ week to 6 months	241	14	1.00 (0.4, 2.7)	0.56 (0.17, 1.81)
After 6 months	45	24	9.2 (3.5,24.2)*	2.7 (1.3, 29.4)*
**Maternal Age**				
15-24	83	12	1	1
25-34	234	26	1.7 (0.6, 4.6)	1.3 (0.2, 6.7)
35+	73	6	1.3 (0.5, 3.4)	0.5 (0.1, 3.0)
**Maternal Education**				
Unable to read & write	211	40	8.4 (2.9, 14.8)*	4.2 (0.8,21.4)
Able to read & write	179	4	1	1
**Place of delivery**				
Health facility	337	14	1	1
Home	53	30	10.6 (3.5,13.8)*	6.1 (2.1, 18.6)*
**Infant ARV prophylaxis**				
Yes	314	14	**1**	1
No	76	30	8.8 (4.4, 17.5)*	6.7 (2.9, 23.1)*
**Feeding practice**				
EBF	374	28	1	1
MF	16	16	12.4 (6.0,18.1)*	8.8 (4.5, 22.8)*
**Infants enrolled for HIV care & support**				
Yes	370	32	1	1
No	20	12	6.9 (3.1, 15.4)*	2.5 (0.6, 10.1)
**Mothers’ treatment**				
ART	245	10	1	1
ARV prophylaxis	110	12	2.6 (1.1, 6.3)*	3.1 (0.7,12.8)
None	15	10	16.0 (6.8,24.9)*	21.6 (14.5, 39.8)*
**Duration of treatment**				
>4 weeks	330	8	1	1
<=4 weeks	25	14	23 (8.8, 39)*	12 (4.2, 45)*

Adjusted for age at DBS tested, maternal age, maternal education, feeding practice, place of delivery and prophylaxis.

*Statistically significant at *P* value less than 0.05 and 95% CI.

However, maternal education and history of enrollment that had significant association in the bivariate analysis were not found to have significant association when adjusted for confounding factors in the final multivariate logistic regression model.

## Discussion

Infants who get infected during pregnancy or while breastfeeding require early HIV diagnosis and timely treatment. As a standard testing mechanism to diagnose HIV infection, DNA/PCR testing services have been expanded in Ethiopia, including Amhara region. Following the expansion of HIV DNA/PCR testing facilities in the region, DBS samples have been collected and tested from all infants born to HIV positive pregnant women. Since the test is strong enough to determine the infection rate with a single test, only the results of PCR for HIV DNA antigen in DBS samples of HEIs were used.

In this study, the prevalence of HIV infection among HIV exposed infants was 10.1%, which was incomparable to the national estimate of 10.9% [3]. This slight difference may be due to the enrollment of a relatively smaller number of samples in our study. Given a number of interventions have been implemented to curb the prevalence of mother to child transmission of HIV, the result in this study could still be considered alarming. Having such a burden during the era of UN’s pledge to eliminate MTCT of HIV by December 31st, 2015 [5], is really a challenge to be on the track and avoid needless deaths of these precious natives of tomorrow. This finding clearly indicated that the progress that has been made to produce an HIV free generation is very slow.

This study demonstrated that a number of factors are related to MTCT of HIV. It is consistent with other studies which revealed that infants having their DBS test done after 6 months of their age were more likely to be HIV positive than infants tested at the age of 6 weeks or between 6 weeks and 6 months [10–13]. This observation may be due to the fact that the older the infants were when they got into health facilities for diagnosis, the more they were likely to be exposed to HIV infection due to the longer time of breast feeding. The risk might have doubled if weaning was not correctly practiced and if a mother is not enrolled in HIV care and support during pregnancy, labor and post-partum period. Maternal ART and infants’ ARV prophylaxis are effective interventions to curb MTCT of HIV during this time [3–5].In addition, early infant diagnosis and enrollment in HIV care and support services would have reduced the chance of mother-to-child-transmission of HIV among HEIs, provided that weaning was correctly practiced.

Infants born at home were 6.7 times more likely to be HIV positive than those born at health facilities. This is due to the fact that HIV positive women attending skilled delivery service would be given antiretroviral treatment or antiretroviral prophylaxis to prevent mother to child transmission of HIV during labor and delivery. Moreover, HIV exposed newborns may have the opportunity to receive ARV prophylaxis immediately, thereby minimizing the risk of acquiring HIV infection during labor and delivery as the highest proportion of newborns are infected during this time. This finding is consistent with studies done in Ethiopia and Tanzania [13–15]. However, in this study a considerable number of pregnant women did not attend skilled delivery care, leaving the devastating effects of HIV to their children. Hence, much concerted efforts are needed to ensure that all pregnant women are receiving skilled delivery services.

Those infants born to mothers who did not receive either ART or ARV prophylaxis were 21.6 times more likely to have a risk of acquiring HIV infection than their counterparts. Taking ART could reduce maternal viral load and subsequent transmission of the virus to the fetuses or newborns. Those mothers who did not have a history of treatment were more likely to get their babies infected than their counterparts. This finding is consistent with studies done elsewhere [13, 15, 16]. Without treatment, up to 40% of babies born to HIV positive mothers will start life being infected, and almost half of these will die before they are two years old [4, 5]. Therefore, there should not be any reluctance or missed opportunity to enroll all HIV positive pregnant women for available HIV care and support services.

Another predictor of mother- to- child transmission of HIV concerned in this study was the use of infanthood ARV prophylaxis. Hence, those infants who did not receive ARV prophylaxis right after birth were at risk of acquiring HIV infection. This might be due to the effect of these drugs to reduce the virus concentration in the newborn’s blood. In fact this was in harmony with results of studies done in Ethiopia and Zambia [6, 8, 10, 13, 14] but not with others [15–17].

Being consistent with studies done in Ethiopia [13] and Kenya [17], infants who received mixed feeding were 9 times more likely to acquire HIV infection than their counterparts. This could be due to contamination of the food during preparation or feeding that might have involved in gastrointestinal infection and laceration which may have led to mucosal barrier breakage and subsequent viral entry to the blood stream and progression of HIV infection [11, 18]. Mothers’ lack of knowledge on the adequacy of breastfeeding to satisfy nutritional demands of their (<6 months) infants might contribute to the observed mixed feeding practice in the region.

There might be missed opportunities such as not giving advice for mothers’ of HEIs on infant feeding option during antenatal follow up visits, delivery or postpartum period. As breast feeding is the most preferred nutritional option for HEIs younger than 6 months [6], we have to make sure that every mother has the right information and means to make appropriate decisions.

Moreover, duration of ART treatment also determines the probability of MTCT of HIV. If pregnant women are on ART treatment for longer than 4 weeks, they would be less likely to transmit HIV to their babies. This observation might be attributed to the effect of the treatment on the viral load and subsequent transmission. This finding is in consistence with other studies [15, 16, 18, 19]. This finding underscores the critical need of earlier diagnosis and timely treatment of HIV among pregnant women.

However, most pregnant women were not coming for ANC at all or were very late, although access to the ANC service was fairly available in the region. The regional health bureau, zonal health departments and district health offices should collaborate and strive to ensure that integrated and continuing education and promotion is given to all pregnant women regarding the need for ANC follow up and use of skilled delivery service including utilization of HIV care and support services. This strategy would eventually ensure elimination of MTCT of HIV in the region.

Unlike other previous studies [8, 13], maternal education and history of enrollment of children for HIV care and support did not show a statistically significant relationship with the outcome variable in the final model. This variation may be due to the difference in sample size, study area, study design, method of analysis or could be also attributed to the quality of data captured.

### Limitation of the study

As this research was conducted using secondary data recorded on paper-based system only, it was impossible to obtain some essential information in the charts, such as partner HIV status, household monthly income, maternal viral load during pregnancy and whether she was infected before or during the last pregnancy.

Some variation in recording of client information among facilities might also have introduced reporting bias. The relatively small sample size might also affect the power of the test. Therefore, generalization of the findings of this study may not be possible. Although this study might suffer from its lower precision, its results would be valuable evidences to evaluate program effectiveness and provide a foundation for future intervention.

## Conclusion

Depending on all the findings deliberated so far, therefore, it is possible to conclude that the prevalence of HIV infection among HEI is strikingly high. Delay in seeking HIV testing services, failure to seek skilled delivery service, mixed infant feeding practice and failure to provide or shorter duration of HIV treatment were the factors that increased the risk of mother- to- child transmission of HIV. Consequently, there is a lot to be done by all the various stakeholders. Health authorities at all levels of the region should work together to ensure that all pregnant women are using ANC and skilled delivery services.

Integrated and audience specific education and promotion on using comprehensive HIV care and support services which include timely HIV testing of HIV exposed infants should be strengthened. Research is needed to explore reasons related to infant mortalities or failures in follow-up of HIV care services in the region. Assessing the risk among clients visiting private providers may also reveal valuable information. Future researchers should consider the use of larger sample size and also the possibility of assessing the factors turned out to be insignificant in this study.

## Abbreviations

AOR: Adjusted Odds ratio
ART: Antiretroviral Therapy
ARV: Antiretroviral
AZT: Zidovudine
CI: Confidence Interval
COR: Crude Odds ratio
DBS: Dried Blood Spot, DNA, Deoxyribonucleic Acid
EBF: Exclusive Breast Feeding
EID: Early Infant Diagnosis
HIV: Human Immunodeficiency Virus
HEI: HIV Exposed Infants
MF: Mixed Feeding
MTCT: Mother to Child Transmission of HIV
NVP: Nevirapin
PCR: Polymerase Chain Reaction
PMTCT: Prevention of Mother to child Transmission
UN: United Nation.
